# The Association of Homocysteine with Metabolic Syndrome in a Community-Dwelling Population: Homocysteine Might Be Concomitant with Metabolic Syndrome

**DOI:** 10.1371/journal.pone.0113148

**Published:** 2014-11-17

**Authors:** Xiaona Wang, Ping Ye, Ruihua Cao, Xu Yang, Wenkai Xiao, Yun Zhang, Yongyi Bai, Hongmei Wu

**Affiliations:** Department of Geriatric Cardiology, Chinese PLA General Hospital, Beijing, China; INRCA, Italy

## Abstract

**Objective:**

Elevated plasma total homocysteine (tHcy) and metabolic syndrome (MetS) are both associated with cardiovascular disease, but the association between tHcy and MetS is not well characterized. The aim of this study was to determine the relationship between tHcy and MetS.

**Methods:**

To further estimate the time-dependent association of tHcy and MetS, we analyzed the tHcy level and MetS in 1499 subjects from a 4.8-year longitudinal study in Beijing, People’s Republic of China.

**Results:**

In multiple linear regression analysis, baseline tHcy levels associated with age, BMI, SBP, DBP, LDL-C and Cr independently over 4.8-years follow-up; age, BMI, SBP, DBP and Cr were found to be associated with tHcy levels independently at the end of follow-up. Logistic regression analysis showed that there was no association between the baseline tHcy level and MetS over the 4.8-year follow-up (odds ratio (OR), 1.32; 95% confidence interval (CI), 0.79–2.19; P = 0.282); rather, there was an association only with hypertension as a MetS component (OR, 1.53; 95% CI, 1.06–2.21; P = 0.024). tHcy levels were associated with MetS at both cross-sectional baseline (OR, 1.38; 95% CI, 1.02–1.88; P = 0.038) and cross-sectional follow-up (OR, 1.60; 95% CI, 1.02–2.50; P = 0.041). The tHcy levels of MetS subjects were higher than those of non-MetS subjects at both cross-sectional baseline (19.35±7.92 µmol/L vs. 17.45±6.70 µmol/L, respectively; P = 0.001) and cross-sectional follow-up (18.95±7.15 µmol/L vs. 17.11±5.98 µmol/L, respectively; P = 0.02).

**Conclusion:**

The tHcy level was not predictive of the incidence of MetS; however, it may be a risk factor for hypertension as a MetS component. Furthermore, tHcy levels were associated with MetS at cross-sectional baseline and follow-up, which suggests that a higher level of tHcy might be concomitant with MetS.

## Introduction

Metabolic syndrome (MetS) is a condition that is characterized by a constellation of metabolic disorders including abdominal obesity, dyslipidemia, hypertension, and hyperglycemia [1]–[3]. The increasing prevalence of MetS is an important public health concern because it appears to directly promote the development of cardiovascular disease (CVD) and type 2 diabetes [4], [5].

Homocysteine (Hcy) is a sulfur-containing amino acid that is formed during the metabolism of methionine, and hyperhomocysteinemia is related to the incidence of cardiovascular and cerebrovascular diseases [6]. Increasing attention has been devoted to the association between the plasma total total Hcy (tHcy) level and MetS, and both positive and negative results have been reported based on cross-sectional studies and longitudinal studies using only baseline measurements of tHcy and MetS [7], [8]. Due to the differences in the reported results, the potential causality in the association between tHcy and MetS is unclear. For example, tHcy may be a risk factor for MetS or simply a concomitant phenomenon of MetS.

Therefore, to better understand the relationship between tHcy and MetS, we analyzed the tHcy -MetS association using both the baseline tHcy level and the follow-up tHcy level in a longitudinal study.

## Methods

### Study population

This was a community-based cohort study of people living in Pingguoyuan area in Beijing, China. After a routine health check-up between September 2007 and January 2009, initially, a total of 1859 permanent subjects were recruited to the study as described previously [9]. 31 subjects with severe systemic diseases such as collagenosis, endocrine and metabolic disease (except diabetes mellitus [DM]), inflammation, neoplastic or severe liver or renal diseases were excluded from the analysis.

Adequate measurement was either not obtained or not attempted in the 86 subjects. Another 37 subjects were excluded because of missing data on tHcy or other measurements. In addition, 25 subjects were excluded because of missing information for multivariable analyses. Thus, 1680 subjects were eligible for analysis.

The study was approved by the ethics committee of People’s Liberation Army General Hospital, and each subject provided written informed consent.

### Follow-up and Outcome Assessment

We prospectively followed this community-based population for the second visit through February 1 to September 30, 2013 by a standard questionnaire and household visit by physician investigators. Demographic information, medical history, blood pressure, and anthropometric measurements were collected. Fasting blood and urine samples were also collected. During a median 4.8 years of follow-up for 1680 subjects, 181 participants were lost and excluded from analysis. Follow-up data were completed for 1499 subjects (follow-up rate 89.2%). All subjects have been followed up for all-cause mortality, CVD mortality, and the development of diabetes from the initial screening to September 30, 2013.

### Clinical data collection

All subjects completed a self-reported standardized questionnaire that included questions about lifestyle factors, prevalent diseases, family history, and medication use. Anthropometrics were evaluated by trained medical doctors. Height was measured in centimeters using a wall-mounted measuring tape, and weight was measured using a digital scale in kilograms (without shoes). Self-reported smoking status was categorized as current, former, or never smoking. Systolic and diastolic blood pressures (SBP and DBP) were measured on the right arm in a sitting position after 5 minutes of rest, and the average SBP and DBP measurements were used for further analysis.

### Biomarker variable determination

All subjects underwent full laboratory evaluation (lipid profile and liver and kidney function indices). Blood samples were collected from subjects between 8 am and 10 am after an overnight fast of at least 12 hours. Plasma aliquots were frozen at −80°C until the assays were performed. Peripheral blood samples were obtained to measure the following parameters: fasting blood glucose (FBG), total cholesterol (TC), triglyceride (TG), low-density lipoprotein cholesterol (LDL-C), high-density lipoprotein cholesterol (HDL-C), uric acid (UA), creatinine (Cr), alanine aminotransferase (ALT), and tHcy. All subjects without a history of DM underwent the standard 75-g oral glucose tolerance test. The estimated glomerular filtration rate (eGFR) was calculated using the following Chronic Kidney Disease Epidemiology Collaboration equations: GFR = 141×min (Scr/κ,1)^α^×max (Scr/κ,1)^−1.209^×0.993^Age^×1.018 [if female]×1.159 [if black], where Scr is plasma creatinine (mg/dL), κ is 0.7 for females and 0.9 for males, α is −0.329 for females and −0.411 for males, min indicates the minimum of Scr/κ or 1, and max indicates the maximum of Scr/κ or 1.

Biochemical variables of all blood specimens were measured with an automated analyzer (Roche Cobas e601, Switzerland) in the same laboratory, following the criteria of the World Health Organization Lipid Reference Laboratories.

### Definition of variables

Hypertension was defined as a systolic BP≥140 mmHg or a diastolic BP≥90 mmHg or the use of antihypertension medication. Smoking status was defined as smoking 1 or more cigarettes per day for at least 1 year. Alcohol users were defined as drinking once a week (white spirit or beer or red wine). Body mass index (BMI) was defined as weight (kilograms) divided by the square of height (meters). Waist circumference was measured at the level of the umbilicus. Exercise status was defined as exercising at least 30 min every day regardless of the type of exercise.

Metabolic syndrome was defined by the Diabetes Branch of the Chinese Medical Association (CDS) [10]. A participant was defined as having MetS if he or she had three or more of the following four medical conditions: (1) overweight or obesity (i.e., BMI≥25.0 kg/m^2^) [11]; (2) hypertension (i.e., SBP≥140 mmHg or DBP≥90 mmHg) or previously diagnosed hypertension; (3) dyslipidemia (i.e., fasting TG≥1.7 mmol/L (150 mg/dl) or fasting HDL-C <0.9 mmol/L (35 mg/dl)); (4) hyperglycemia defined as either FPG≥6.1 mmol/L (110 mg/dl) or 2 h postprandial glucose ≥7.8 mmol/L (140 mg/dl) or as previously diagnosed as hyperglycemia.

### Statistical analyses

tHcy levels were categorized as high (≥15 µmol/L) or normal (<15 µmol/L) [12]. tHcy and other parameters (TG, Cr, eGFR) were normalized by natural logarithm transformation. Baseline continuous variables are presented as the mean ± standard deviation (SD) or median (with interquartile range) and were analyzed with Student’s t-tests; baseline dichotomous variables are presented as numbers and percentages and were compared using the x^2^ test.

A Pearson regression analysis, a stepwise multivariate linear regression analysis and a multicollinearity analysis were performed to evaluate the associations between baseline tHcy and other parameters over 4.8-years follow-up, tHcy and other parameters at the end of follow up.

In addition, a forward stepwise multivariate logistic regression analysis was performed to obtain the odds ratios (OR) and 95% confidence intervals (CI). We investigated the association of baseline tHcy with MetS over 4.8-year follow up, tHcy and MetS at both cross-sectional baseline and follow-up. Regression models were adjusted for age and sex as the independent variable (Model 1) and additionally adjusted for smoking, alcohol use (g/day), TC, LDL-C, ALT, UA, Cr and eGFR as the independent variables (Model 2).

The difference of tHcy levels between MetS subjects and non-MetS subjects at both cross-sectional baseline and follow-up were analyzed with Student’s t-tests.

All analyses were conducted using SPSS software for Windows, version 13.0 (SPSS, Chicago, IL, USA). P values <0.05 were considered statistically significant.

## Results

### Clinical characteristics of the subjects categorized by tHcy level

The baseline characteristics of the study subjects are summarized in Table 1. The mean age of the participants at baseline was 61.40 years, and 57.97% were women. The baseline prevalences of MetS, DM, and CVD were 32.69% (490), 20.94% (314), and 12.27% (184), respectively.

**Table 1:** Clinical characteristics of the subjects categorized by tHcy level at baseline.

Variable	All subjects	tHcy<15 µmol/L	Hcy ≥15 µmol/L	P-value
No of subjects	1499	468	1031	
Age (y)	61.40±11.4	57.71±9.91	64.32±12.35	<0.001
Women [n (%)]	869 (59.97)	338 (72.22)	531 (51.50)	<0.001
BMI (kg/m^2^)	25.41±3.32	25.22±3.36	25.72±3.39	0.015
TG (mmol/l)	1.90±1.24	1.73±1.17	1.85±1.21	0.691
TC (mmol/l)	5.03±0.93	5.06±0.92	5.01±0.91	0.221
HDL-C (mmol/l)	1.38±0.36	1.47±0.31	1.31±0.27	<0.001
LDL-C (mmol/l)	2.91±0.71	2.93±0.71	2.96±0.72	0.479
tHcy (µmol/L)	18.41±8.23	12.04±2.25	21.80±7.38	<0.001
SBP (mmHg)	128.7±17.7	125.25±18.39	132.23±18.60	<0.001
DBP (mmHg)	76.9±10.2	76.34±10.01	77.05±10.98	0.314
FBG (mmol/L)	5.39±1.65	5.51±1.83	5.26±1.61	0.006
2-HpBG (mmol/L)	7.03±4.21	6.89±4.27	7.11±4.23	0.573
eGFR (ml/min/1.73 m^2^)	94.2±14.3	77.13±14.98	108.67±13.14	<0.001
UA (mmol/l)	292.37±69.62	275.89±68.99	317.68±70.01	<0.001
Cr (mmol/l)	64.15±19.94	60.92±16.01	69.50±18.36	<0.001
Current smoking [n (%)]	394 (26.28)	90 (19.23)	304 (29.49)	<0.001
Current alcohol use [n (%)]	284 (18.94)	61 (13.03)	223 (21.63)	<0.001
Diabetes [n (%)]	314 (20.94)	108 (23.07)	206 (19.98)	0.172
History of CVD [n (%)]	184 (12.27)	45 (9.26)	139 (13.48)	0.034
MetS [n (%)]	490 (32.69)	131 (27.99)	359 (34.82)	<0.001
Hypertension [n (%)]	783 (52.23)	201 (42.94)	582 (56.45)	<0.001

**Notes:** Continuous variables (Age, BMI, TG, TC, HDL-C, LDL-C, tHcy, SBP, DBP, 2h-PBG, eGFR, UA, Cr) were expressed as mean (±SD) or median (interquartile range), and categorical variables (Women, Current smoking, Current alcohol use, Diabetes, CVD, MetS and hypertension) were expressed as counts and percentages.

**Abbreviations:** BMI, body mass index; TC, total cholesterol; TG, triglyceride; LDL-C, low density lipoprotein cholesterol; HDL-C, high density lipoprotein cholesterol; tHcy, total homocysteine; UA, uric acid; Cr, Creatinine; FBG, fasting blood glucose; 2-HpBG, 2-hour postprandial blood sugar; SBP, systolic blood pressure; DBP, diastolic blood pressure; eGFR, estimated glomerular filtration rate; MetS, metabolic syndrome; CVD, cardiovascular disease.

### Linear regression analysis of tHcy levels and other parameters

The results of linear regression analysis of tHcy levels and other parameters are shown in table 2. At follow-up cross-section, age, BMI, waist circumstance (Wc), SBP, DBP, Ua, Cr were associated with tHcy positively in Pearson correlation analysis; in multiple linear regression analysis, age, BMI, SBP, DBP and Cr were found to be associated with tHcy levels independently. Over 4.8-year follow-up, baseline tHcy was positively associated with follow-up age, BMI, Wc, SBP, DBP, LDL-C, Ua, Cr, but inversely associated with HDL-C in Pearson correlation analysis; in multiple linear regression analysis, follow-up age, BMI, SBP, DBP, LDL-C and Cr were found to be independently associated with baseline tHcy levels. All the Variance Inflation Factor (VIF) is less than 10, and there was no multicollinearity.

**Table 2:** Univariate, Stepwise multivariate linear regression and Multicollinearity analysis of tHcy levels and other parameters.

Measurement of tHcy*										
Baseline tHcy* predict other parameters over 4.8 years follow-up						tHcy* and other parameters at the end of follow-up				
	r	P	R	p	VIF	r	P	R	P	VIF
Sex	−0.191	<0.001	−0.067	0.005		−0.126	<0.001	−0.095	0.007	
Age	0.268	<0.001	0.216	<0.001	1.229	0.265	<0.001	0.263	<0.001	1.256
BMI	0.054	0.004	0.148	0.001	2.527	0.024	0.391	0.009	0.013	2.631
Wc	0.215	<0.001	−0.201	<0.001	2.739	0.207	<0.001	−0.190	<0.001	2.906
SBP	0.063	<0.001	0.023	0.029	1.203	0.125	<0.001	0.027	0.389	1.213
DBP	0.033	0.007	0.050	0.034	1.149	0.799	0.009	0.035	0.252	1.122
TG*	0.027	0.386	0.069	0.046	1.517	−0.011	0.705	0.054	0.111	1.538
TC	−0.032	0.263	0.053	0.223	2.552	−0.069	0.015	−0.075	0.087	2.526
HDL	−0.171	<0.001	−0.123	0.005	2.620	−0.056	0.051	−0.042	0.325	2.570
LDL	0.111	<0.001	0.071	0.019	3.550	0.027	0.344	0.063	0.227	3.538
UA	0.269	<0.001	0.177	<0.047	1.449	0.216	<0.001	0.126	0.051	1.449
Cr*	0.492	<0.001	0.507	<0.001	2.535	0.365	<0.001	0.068	<0.001	2.483
FBG	−0.071	0.014	−0.070	0.019	1.078	−0.004	0.901	−0.025	0.390	1.083
e-GFR*	−0.189	<0.001	−0.082	0.007	1.545	−0.238	<0.001	−0.135	<0.001	1.502

**Note:** r: Pearson correlation coefficient; R: Stepwise multiple linear correlation coefficient; VIF: Variance Inflation Factor.

*: after natural logarithm transformed.

**Abbreviations:** tHcy, total homocysteine; BMI, body-mass index; Wc, waist circumstance; SBP, systolic blood pressure; DBP, diastolic blood pressure; TC, total cholesterol; TG, triglyceride; LDL-C, low density lipoprotein cholesterol; HDL-C, high density lipoprotein cholesterol; UA, uric acid; Cr, Creatinine; FBG, fasting blood glucose; eGFR, estimated glomerular filtration rate.

### Baseline tHcy level and the incidence of MetS over 4.8 years of follow-up

Among 1009 individuals who were free of MetS at baseline, 169 had developed MetS at the 4.8-year follow-up. The results for individuals in the overall sample are shown in Table 3. There was an association only between baseline tHcy and hypertension as a MetS component (OR, 1.53; 95% CI, 1.06–2.21; P = 0.024). The baseline tHcy was associated with the occurrence of MetS (OR, 1.61; 95% CI, 1.05–2.48; P = 0.039) in the unadjusted model, but the association disappeared when the models were additionally adjusted for age, sex, smoking, alcohol use, TC, LDL-C, ALT, UA, Cr, and eGFR (OR 1.32; 95% CI, 0.79–2.19; P = 0.282).

**Table 3:** Baseline tHcy level and the incident of MetS over 4.8 years of follow-up.

	Levels of tHcy	
Incident of MetS	OR (95% CI)	P-value
Unadjusted	1.61 (1.05–2.48)	0.029
Model 1	1.35 (0.85–2.15)	0.203
Model 2	1.32 (0.79–2.19)	0.282
BMI^a^	1.12 (0.78–1.62)	0.548
Hypertension^a^	1.53 (1.06–2.21)	0.024
Hyperglycemia^a^	0.69 (0.43–1.09)	0.112
Dyslipidemia^a^	1.08 (0.71–1.63)	0.716

**Notes:** Levels of tHcy were Ln transformed to normalize their distributions. Data were presented as ORs (per SD increase in LntHcy levels) and corresponding 95% CIs. In the logistic regression model, MetS was treated as the dependent variable (MetS versus NonMetS). Model1: adjusted for age, sex; Model 2: adjusted for age, sex, smoking, alcohol use, TC, LDL-C, UA, Cr, and eGFR. a: adjusted for age, sex, smoking, alcohol use, UA, Cr, and eGFR. BMI: ≥25.0 kg/M^2^; Hypertension: systolic blood pressure (SBP) ≥140 mmHg, or diastolic blood pressure (DBP) ≥90 mmHg, or previously diagnosed as hypertension; Hyperglycemia: fasting blood-glucose (FPG) ≥6.1 mmol/L (110 mg/dl), or 2 h postprandial glucose (PG) ≥7.8 mmol/L (140 mg/dl), or previously diagnosed as hyperglycemia; Dyslipidemia: fasting triglycerides (TG) ≥1.7 mmol/L (150 mg/dl), or fasting HDL-C<0.9 mmol/L (35 mg/dl).

**Abbreviations:** BMI, body mass index; TC, total cholesterol; LDL-C, low density lipoprotein cholesterol; tHcy, total homocysteine; UA, uric acid; Cr, Creatinine; eGFR, estimated glomerular filtration rate; MetS, metabolic syndrome.

### tHcy level and MetS at cross-sectional baseline

**Figure 1:**
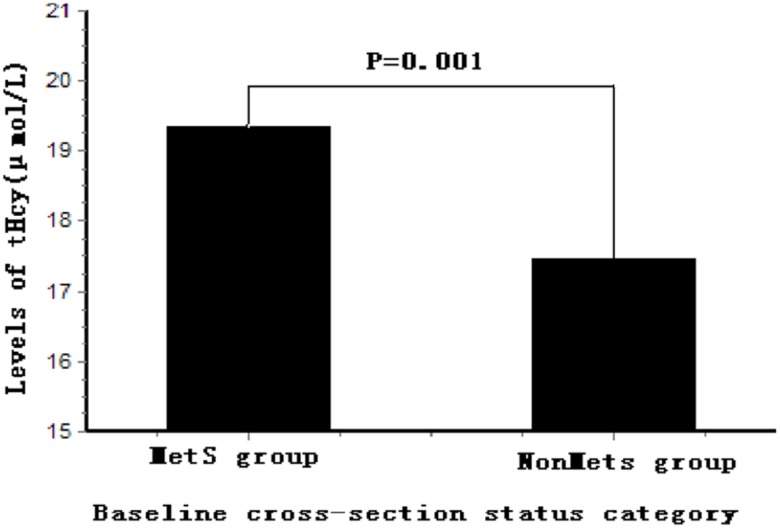
Comparison of serum tHcy levels between MetS group and NonMetS group at baseline MetS status. The levels of tHcy in the MetS group were higher than that in NonMetS group (P = 0.001) at baseline cross-sectional status. Levels of tHcy were Ln transformed to normalize their distributions. tHcy, total homocysteine; MetS, metabolic syndrome.

In addition, we analyzed the cross-sectional baseline tHcy and MetS, and the results are shown in Table 4. There was a significant association between tHcy and MetS (OR, 1.50; 95% CI, 1.16–1.93; P<0.001), and the result did not change appreciably when the models were additionally adjusted for age, sex, smoking, alcohol use, TC, LDL-C, ALT, UA, Cr, and eGFR (OR, 1.38; 95% CI, 1.02–1.88; P = 0.038). BMI was significantly associated with tHcy after adjusting for age, sex, smoking, alcohol use, ALT, UA, Cr, and eGFR (OR, 1.29; 95% CI, 1.09–1.62; P = 0.002). The tHcy level of MetS subjects at baseline was higher than that of non-MetS subjects (19.35±7.92 µmol/L vs. 17.45±6.70 µmol/L, respectively; P = 0.001) (Figure 1).

**Table 4:** tHcy level and MetS at cross-sectional baseline.

	Levels of tHcy	
Incident of MetS	OR (95% CI)	P-value
Unadjusted	1.50 (1.16–1.93)	<0.001
Model 1	1.49 (1.10–1.73)	0.005
Model 2	1.38 (1.02–1.88)	0.038
BMI^a^	1.29 (1.09–1.62)	0.002
Hypertension^a^	1.35 (0.89–1.21)	0.428
Hyperglycemia^a^	0.87 (0.73–1.43)	0.513
Dyslipidemia^a^	1.16 (0.93–1.63)	0.108

**Notes:** Levels of tHcy were Ln transformed to normalize their distributions. Data were presented as ORs (per SD increase in LntHcy level) and corresponding 95% CIs. In the logistic regression model, MetS was treated as the dependent variable (MetS versus NonMetS). Model 1: adjusted for age, sex; Model 2: adjusted for age, sex, smoking, alcohol use, TC, LDL-C, UA, Cr, and eGFR; a: adjusted for age, sex, smoking, alcohol use, UA, Cr, and eGFR. BMI: ≥25.0 kg/M^2^; Hypertension: systolic blood pressure (SBP) ≥140 mmHg, or diastolic blood pressure (DBP) ≥90 mmHg, or previously diagnosed as hypertension; Hyperglycemia: fasting blood-glucose (FPG) ≥6.1 mmol/L (110 mg/dl), or 2 h postprandial glucose (PG) ≥7.8 mmol/L (140 mg/dl), or previously diagnosed as hyperglycemia; Dyslipidemia: fasting triglycerides (TG) ≥1.7 mmol/L (150 mg/dl), or fasting HDL-C <0.9 mmol/L (35 mg/dl).

**Abbreviations:** BMI, body mass index; TC, total cholesterol; LDL-C, low density lipoprotein cholesterol; tHcy, total homocysteine; UA, uric acid; Cr, Creatinine; eGFR, estimated glomerular filtration rate; MetS, metabolic syndrome.

### tHcy level and the incidence of MetS at cross-sectional follow-up

**Figure 2:**
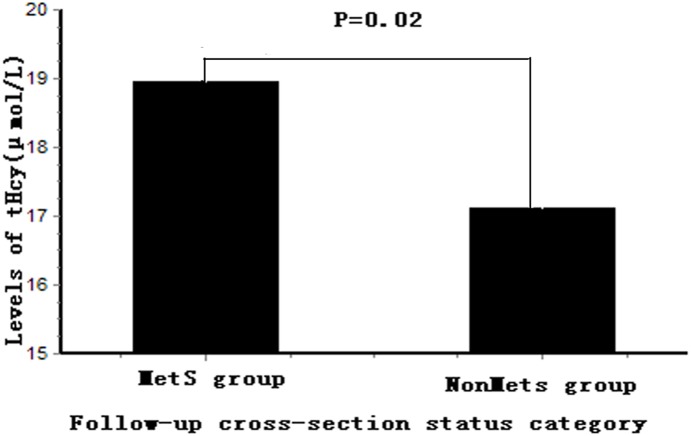
Comparison of serum tHcy levels between MetS group and NonMetS group at follow-up MetS status. The levels of tHcy in the MetS group were higher than that in NonMetS group (P = 0.02) at follow-up cross-sectional status**.** Levels of tHcy were Ln transformed to normalize their distributions. tHcy, total homocysteine; MetS, metabolic syndrome.

The association between tHcy and MetS at cross-sectional follow-up is shown in Table 5. There was a significant association between tHcy and MetS (OR, 1.75; 95% CI, 1.22–2.52; P = 0.002), and the result did not change appreciably when the models were additionally adjusted for age, sex, smoking, alcohol use (g/day), TC, LDL-C, ALT, UA, Cr, and eGFR (OR, 1.60; 95% CI, 1.02–2.50; P = 0.041). BMI was still significantly associated with tHcy after adjusting for age, sex, smoking, alcohol use (g/day), ALT, UA, Cr, and eGFR (OR, 1.87; 95% CI, 1.29–2.69; P = 0.001). The tHcy level at cross-sectional follow-up in MetS subjects was higher than that of non-MetS subjects (18.95±7.15 µmol/L vs. 17.11±5.98 µmol/L, respectively; P = 0.02) (Figure 2).

**Table 5:** tHcy level and the incident of MetS at cross-sectional follow-up.

	Levels of tHcy	
Incident of MetS	OR (95% CI)	P-value
Unadjusted	1.75 (1.22–2.52)	0.002
Model 1	1.54 (1.04–2.27)	0.029
Model 2	1.60 (1.02–2.50)	0.041
BMI^a^	1.87 (1.29–2.69)	0.001
Hypertension^a^	1.34 (0.96–1.92)	0.083
Hyperglycemia^a^	0.87 (0.60–1.25)	0.437
Dyslipidemia^a^	0.79 (0.53–1.20)	0.281

**Notes:** Levels of tHcy were Ln transformed to normalize their distributions. Data were presented as ORs (per SD increase in LntHcy level) and corresponding 95% CIs. In the logistic regression model, MetS was treated as the dependent variable (MetS versus NonMetS). Model1: adjust for age, sex; Model2: adjust for age, sex, smoking, alcohol use, TC, LDL-C, UA, Cr, and eGFR; a: adjust for age, sex, smoking, alcohol use, UA, Cr, and eGFR. BMI: ≥25.0 kg/M^2^; Hypertension: systolic blood pressure (SBP) ≥140 mmHg, or diastolic blood pressure (DBP) ≥90 mmHg, or previously diagnosed as hypertension; Hyperglycemia: fasting blood-glucose (FPG) ≥6.1 mmol/L (110 mg/dl), or 2 h postprandial glucose (PG) ≥7.8 mmol/L (140 mg/dl), or previously diagnosed as hyperglycemia; Dyslipidemia: fasting triglycerides (TG) ≥1.7 mmol/L (150 mg/dl), or fasting HDL-C<0.9 mmol/L (35 mg/dl).

**Abbreviations:** BMI, body mass index; TC, total cholesterol; LDL-C, low density lipoprotein cholesterol; tHcy, total homocysteine; UA, uric acid; Cr, Creatinine; eGFR, estimated glomerular filtration rate; MetS, metabolic syndrome.

## Discussion

In the present study, we found no association between the baseline tHcy level and the incidence of MetS over 4.8 years of follow-up; however, we identified an association with hypertension as a MetS component. We demonstrated an association between tHcy levels and MetS at cross-sectional baseline and follow-up, which was mainly strengthened by BMI but not by other MetS components. These findings suggest that a higher level of tHcy might be concomitant with MetS, especially in obese subjects.

The association between tHcy and MetS is not well characterized, and studies investigating this association have shown conflicting results. Hajer found that the tHcy level was higher in the metabolic syndrome group than in the control group and that it increased with the presence of its components [13]. In contrast, Garcin found that tHcy is not associated with metabolic syndrome [14], while other studies have described an association between tHcy and MetS components but not between tHcy and MetS [15], [16]. These discrepancies may be attributed to the following factors. First, the study populations were different. Plasma total tHcy levels vary by age and have significant ethnic- and gender-dependent differences [17]–[19]. The tHcy level is higher in China than in other countries, and it is much higher in people of northern China than in those living in southern areas because of the lower intake of high-folate-containing green leafy vegetables among northerners [20]. In the present study, the average tHcy level was 18.28±7.65 (µmol/L) in subjects from Beijing, which is in northern China. Second, most previously published studies focused on the relationship between the baseline tHcy level and MetS. In this study, we analyzed the tHcy-MetS association using both the baseline tHcy level and the follow-up tHcy level through a longitudinal study.

We have reported the association between tHcy level and MetS before [21], which was a cross-sectional study. Because of the cross-sectional design and its inherent limitations, the previous study cannot determine causal relationships between the tHcy and MetS. Accordingly, we designed this longitudinal study, which not only tracks each participant’s outcome (e.g., MetS) but also repeatedly measures risk factors that would change with time (e.g., tHcy). In the present study, we spended a median 4.8 years of follow-up. Furthermore, the present study used the Diabetes Branch of the Chinese Medical Association criteria which was more suitable for Chinese [10], while the previous related manuscript used NCEP-ATPIII criteria.

The main finding of this study was that a higher level of tHcy might be concomitant with MetS. The key link between tHcy and MetS lies in insulin resistance, which affects the tHcy level in MetS [22]. The Framingham Study found that elevated levels of fasting insulin are significantly associated with fasting homocysteine, even after adjustment for several important confounders [23]. Another clinical study found that the change in the tHcy level during hyperinsulinemia is correlated with insulin sensitivity [24]. Insulin resistance elevates tHcy along with reciprocal changes in two key enzymes in tHcy metabolism: cystathionine β-synthase (CβS) and methylenetetrahydrofolate reductase [25]. Additionally, hyperhomocysteinemia can further induce insulin resistance [26], leading to a worsening of both conditions. Further, insulin resistance induces vascular injury and endothelial dysfunction, which may reduce excretion of tHcy by damaging glomerular filtration function [14].

The second important finding of this study is that the association between tHcy and MetS was mainly strengthened by BMI at cross-sectional baseline and follow-up. Obesity is usually associated with a high intake of fats, which are present in foods such as red meat and chicken and increase the plasma total tHcy level by downregulating hepatic CβS and cystathionine γ-lyase activity [27]. Obesity also induces insulin resistance, which is a major factor associated with hyperhomocysteinemia [28].

Finally, this study found that the baseline tHcy level was not predictive of the incidence of MetS; however, it was associated with hypertension (a MetS component). Hyperhomocysteinemia may damage both conduit and resistance vessel endothelial function, which manifests as a reduction of vessel flexibility and dilatation [29], thus promoting the development of hypertension. Tayama found that a higher plasma total homocysteine concentration is associated with increased systemic arterial stiffness, which may enhance blood pressure reactivity to stress in hypertensive patients [30].

There are several strengths to our study. First, this is the first time that the relationship between tHcy and MetS has been studied in China. Second, this was a longitudinal study in which the association of tHcy with MetS was analyzed using baseline and follow-up data; thus, it differs from cross-sectional studies or cohort studies that describe only baseline tHcy measurements.

The present study has limitations. First, the study was performed on Chinese residents from two communities in Beijing; thus, the results may not represent Chinese individuals from other areas. Second, 181 subjects (10.7%) were lost to follow-up. This loss is an unavoidable limitation of epidemiological studies that may be biased toward the null hypothesis due to the loss of cases that presumably had more extreme values for the analyzed variables. Third, folate and Vit B12 level was not measured in this study which may be helpful for explaining the relationship between tHcy and MetS. Fourth, although this is a longitudinal study, the methods used in the analysis of the results does not allow a speculation for causality between tHcy levels and MetS or its components.

## Conclusion

In conclusion, tHcy levels have no predictive effect on the incidence of MetS but may be a risk factor for hypertension (a MetS component) in this longitudinal study. tHcy levels were cross sectionally associated with MetS at baseline and follow-up, and a higher level of tHcy might be concomitant with MetS.
